# Optimized Intelligent Localization Through Mathematical Modeling and Crow Search Algorithms

**DOI:** 10.3390/s25154804

**Published:** 2025-08-05

**Authors:** Tamer Ramadan Badawy, Nesreen I. Ziedan

**Affiliations:** 1 Communications and Computer Engineering Department, Misr Higher Institute for Engineering and Technology, Mansoura 7651012, Egypt; 2 Computer and Systems Engineering Department, Faculty of Engineering, Zagazig University, Zagazig 7120001, Egypt; ziedan@outlook.com

**Keywords:** indoor localization, 2D position estimation, swarm intelligence, Crow Search Algorithm, mobile localization, indoor localization, position estimation

## Abstract

Localization has emerged as a critical problem over the past decades, with diverse techniques developed to address robot and mobile localization challenges across varied domains. However, existing localization methods still fall short of achieving the precision needed for certain high-demand applications. The proposed algorithm is designed to enhance localization accuracy by integrating mathematical modeling with the Crow Search Algorithm (CSA). The objective is to identify the most probable position within a designated search space. Anchored by a network of fixed points, the search area is initially defined. A mathematical approach is then applied to reduce this area by calculating the intersections between circles centered at each anchor point. Within this reduced area, an array of candidate points are selected, and their centroid is computed to serve as an initial estimate. The modified CSA iteratively improves upon this estimate by emulating the natural behavior of crows, updating its variables to converge on the optimal position. Experimental evaluations, conducted on both real and simulated datasets, demonstrate that the proposed algorithm leads to a better localization accuracy than existing methods. The proposed methodology achieves a significant accuracy improvement with an accuracy of 98%. These results confirm the effectiveness of our approach for applications that require high precision with minimal infrastructure and low computational complexity.

## 1. Introduction

The main objective of indoor 2D localization is to accurately obtain the position of an object on a plane area by obtaining information from sensors fixed at reference points known as anchors and taking into account the percentage of error obtained from these sensors. Additionally, it should be low-cost, low-complexity, and require minimal infrastructure, as well as take into account noisy environments that affect the performance of the sensors.

Many methodologies have been proposed, from mathematical models using statistics and probability to learning methods and optimization algorithms. These methods vary significantly in terms of computational requirements, hardware, complexity, dependencies, and adaptability to real-time systems.

Indoor localization is the main problem in a wide range of applications like mobile robotics, wireless sensor networks, and some industrial applications. Two-dimensional localization is still a critical research area, specifically indoors and in closed areas, where the global navigation satellite system (GNSS) [1,2,3] is not applicable or is unavailable due to having a weak signal. Processing GNSS signals under these conditions often requires advanced algorithms [4,5], which can be costly and consume significant battery power in mobile devices [6,7,8]. Consequently, numerous GNSS alternative techniques have been developed to tackle localization challenges, enhancing the positioning and tracking of mobile devices, pedestrians, vehicles, and robots [9,10].

Traditional localization techniques typically rely on distance, angle, or received signal strength (RSS), or combine these metrics in hybrid approaches [11,12,13]. RSS-based methods are particularly appealing in robotics due to their low costs, reduced processing time, and lack of hardware requirements. Conversely, methods such as Time of Arrival (TOA) [14] and Angle of Arrival (AOA) [15] require specialized equipment for precise time and angle measurements. RSS-based localization is prevalent in some applications such as cost-sensitive robotics [16].

Localization commonly involves gathering measurements such as ranges or angles, followed by geometric methods like trilateration [17] or triangulation [18] to estimate the target position. Localization approaches can be broadly categorized as range-based or range-free. Range-based techniques rely on polar coordinates derived from distance and angle measurements, while range-free techniques use centroid- and hop-based algorithms. Typically, range-based methods provide a higher accuracy but additional complexity [12,13].

RSS-based localization requires anchor nodes positioned at known positions. This process involves three steps: (1) receiving signal power from each anchor, (2) estimating distances using models such as distance loss [19,20,21] or polynomial fitting [22,23], and (3) calculating the positions using methods like maximum likelihood estimation (MLE) [24,25], two-step estimation techniques [26], or environmental adaptation algorithms [27]. However, the accuracy and reliability of RSS localization often suffer due to multipath interference, noise, and other environmental effects.

Mathematical models [15,19] can enhance localization by leveraging anchor nodes to define measurement-based circles around each anchor. In a two-dimensional setting, these circles ideally intersect at a single point, representing the target position. In practice, due to measurement inaccuracies and suboptimal anchor arrangements, the intersections form an area rather than a distinct point, transforming the problem into finding an optimal point within this region to minimize error [20]. Extensive research has addressed optimal anchor placement to improve localization precision and cost-efficiency [14,16].

Among mathematical approaches, the min–max algorithm [21,28] is widely utilized for its simplicity and computational efficiency. By approximating circles as squares centered at anchor points, it finds a point in the intersection region with minimal error. Some adaptations assign weights to anchors based on signal quality or proximity, further refining accuracy [29]. Despite these improvements, mathematical methods alone often fall short of high-precision localization, especially under significant measurement noise. They do, however, provide a basis for more sophisticated optimization algorithms.

Optimization techniques [30,31,32] are essential for localization, as they reformulate the problem to find a position that minimizes the discrepancy between estimated and actual distances from anchors. Broadly, optimization methods are classified as traditional or metaheuristic. Traditional optimization techniques, including gradient descent [30], the Newton–Raphson method [31], and linear programming [32], require precise mathematical models and are generally effective for convex problems. However, localization is inherently non-linear, non-convex, and multi-modal due to environmental noise and measurement errors, making these methods prone to local minima and high computational costs.

Metaheuristic algorithms address these limitations with high-level strategies that deliver near-optimal solutions in complex optimization scenarios within reasonable timeframes. These include Genetic Algorithms (GAs), Simulated Annealing (SA), Particle Swarm Optimization (PSO), and Ant Colony Optimization (ACO), which model natural processes to efficiently search vast solution spaces and overcome local minima [33]. Swarm intelligence, a subset of metaheuristics, has gained popularity in localization optimization. PSO, for example, minimizes measurement error but can be computationally demanding in large search spaces and may converge prematurely.

For instance, WiFi-based methods such as Received Signal Strength Indicator (RSSI) fingerprinting have been effective in applications like parking vacancy detection [34]. Infrared-based localization systems utilize angle-of-arrival sensors to achieve a high accuracy in constrained indoor environments [35]. Bluetooth Low Energy (BLE) technology has also been widely adopted, particularly in museums and retail applications, due to its low costs and ease of deployment [36]. Moreover, RF-based localization approaches leveraging smartphone sensors and wireless signals have been surveyed and applied in real-world scenarios [37]. These diverse techniques highlight the extensive efforts undertaken in the field, yet challenges remain in balancing accuracy, cost, infrastructure requirements, and robustness against environmental noise. As such, there is a continuous need for novel approaches that can improve localization performance, especially in complex or resource-constrained settings.

In this paper, ultrasonic sensors are used, which measure distance by using ultrasonic waves. The sensor head sends an ultrasonic wave and receives a wave that is reflected back from the target. Ultrasonic sensors measure the distance to the target by measuring the time between the emission and reception.

Ultrasonic sensors work by sending out a sound wave at a frequency above the range of human hearing. Ultrasound is reliable in any lighting environment and can be used in indoor localization problems. Ultrasonic sensors [38] in robots can handle collision avoidance and can be moved around frequently.

An ultrasonic sensor has the following parameters [38]: operating frequency: typically 40 kHz, measurement range: 2 cm–400 cm, accuracy: ±3 mm under standard conditions, resolution: 1 cm, and update rate: up to 20 Hz.

The distance can be calculated by $d=\left(\frac{1}{2}*T*C\right),$ where *T* is the time between the emission and reception, and *C* is the sonic speed.

In addition to traditional localization techniques, speech-based localization [39] using microphone arrays has gained significant attention over the past two decades as a viable acoustic-based positioning approach. These systems utilize time difference of arrival (TDOA) or angle of arrival (AOA) measurements of speech or ambient sound signals captured by spatially distributed microphones to estimate the position of a sound source. Techniques in this domain often rely on beamforming, cross-correlation, and subspace methods to enhance localization accuracy in reverberant environments.

One of the core challenges in indoor localization, especially with non-RF and non-vision-based systems, lies in the signal processing of raw sensor data. In our ultrasonic-based approach, signal degradation due to multipath propagation, environmental noise, and surface reflections introduces uncertainty in time-of-flight (ToF) measurements. These non-linearities and disturbances often violate the assumptions of linear models (LMs) or conventional least squares (LS) techniques, including recursive LS (RLS), which presume Gaussian noise and well-defined observation models. To mitigate these limitations, we propose the use of a Modified Crow Search Algorithm (MCSA), which can operate efficiently even with noisy, partial, or imprecise measurements, without requiring a strict analytical model.

Visual localization approaches [40] such as PNeRFLoc and NeRF-Loc have introduced neural radiance fields (NeRFs) to represent spatial information in a more expressive way. These methods achieve a high accuracy (approximately to the sub-meter level) by learning scene geometry and appearance jointly, making them particularly effective for dynamic and visually complex environments. However, their real-time application is still limited by their significant computational demands. Similarly, LASER (LAtent SpacE Rendering) [41] uses deep feature rendering to improve localization performance in scenes with repetitive structures or sparse textures. It offers a competitive accuracy but also suffers from latency and hardware dependence.

In contrast, traditional LiDAR-based techniques [42] remain robust and reliable, especially in structured indoor environments. The method proposed used correlative scan matching with 2D LiDAR, achieving real-time performance and high localization fidelity, although it relies on precise sensor calibration.

Alternative approaches based on magnetic field [43] mapping or smartphone sensors offer solutions that are more scalable. For instance, magnetic field-based localization has been shown to be feasible without external infrastructure, though its accuracy remains modest. Similarly, smartphone sensor fusion methods [44] enable device-based localization at the cost of precision.

Although the Crow Search Algorithm (CSA) has been previously proposed and applied in various optimization contexts, the novelty of this research lies in the adaptation and enhancement of the CSA to address the specific challenges of 2D indoor localization. In its standard form, the CSA lacks the spatial constraints and domain-specific modifications required for precise and efficient location estimation within indoor environments. To overcome these limitations, this study presents a Modified Crow Search Algorithm (MCSA) that is customized and hybridized through the integration of mathematical modeling and spatial heuristics.

The proposed approach begins by mathematically reducing the search area using two newly developed strategies: the square-based approach (SBA) and the circle-based approach (CBA). These methods utilize the geometric relationships among reference anchor points to define a smaller, more relevant region in which the localization process is performed. This significantly limits the solution space and enables the faster convergence of the optimization process. Within this reduced area, a set of candidate positions is generated, and the centroid of these points is calculated to serve as the initial input for the swarm search. This initialization step provides the algorithm with a closer approximation to the true location and increases the likelihood of identifying the global minimum with fewer iterations.

Moreover, the behavioral rules of the CSA have been modified to suit the demands of localization tasks. The memory update mechanism and position update strategies of the crows have been adapted to reflect more accurate spatial exploration, while the fitness function has been tailored to directly minimize the localization error. This adaptation ensures that the optimization process is not only generic but also aligned with the physical constraints and performance requirements of real-world localization scenarios.

Overall, the novelty of this work lies in its domain-specific customization of CSA, the introduction of a mathematically constrained search strategy, and the centroid-based intelligent initialization. The hybrid model achieves improved localization accuracy and robustness, as demonstrated through both simulation and real-world experimental results. Specifically, the proposed method reaches up to a 98% localization accuracy while maintaining low computational complexity and minimal infrastructure requirements. These findings position the approach as a strong candidate for practical indoor localization applications in noisy and constrained environments.

This paper presents a novel localization algorithm that combines a mathematical modeling approach with the Crow Search Algorithm (CSA), a swarm-based optimization technique. The goal is to improve the positioning accuracy of target nodes while reducing computational time. To achieve this, the algorithm initially narrows down the search area by calculating the intersection region of measured ranges from multiple anchor points, utilizing either a square-based approach (SBA) or a circular-based approach (CBA). The centroid of this intersection region provides an optimal starting point for the CSA, enhancing convergence and accuracy. By merging the straightforward search space reduction offered by mathematical methods with the adaptive efficiency of the CSA, the proposed approach achieves a higher localization accuracy with a reduced computational load.

The main contributions of this paper are as follows:

- Integration of mathematical modeling with metaheuristic optimization: A novel hybrid framework that uses centroid-based initialization from mathematical techniques (the SBA and CBA) is shown to improve the convergence and accuracy of the Crow Search Algorithm (CSA) for indoor localization;
- Enhanced accuracy with reduced complexity: The proposed model was demonstrated to achieve a 98% localization accuracy with fewer anchors and lower computational complexity compared to traditional methods;
- Adaptive search space reduction: The mathematical approaches efficiently minimize the search area and define robust starting points, reducing the reliance on random initialization and improving algorithm stability;
- Robustness in real-world noisy conditions: An evaluation using both real-world and simulated data shows the system maintains high performance under signal noise and anchor drop-out scenarios.

The remainder of this paper is organized as follows. Section 2 introduces the mathematical approach for defining the intersection area, detailing both the square-based (SBA) and circular-based (CBA) methods. Section 3 provides an in-depth description of the CSA. In Section 4, the proposed MCSA is introduced in conjunction with the arithmetic approaches. In Section 5, the performance is evaluated using both real and simulated datasets. Finally, Section 6 presents the conclusions.

## 2. Mathematical Approaches

The mathematical modeling proposed in this work consists of two geometric strategies, the square-based approach (SBA) and circle-based approach (CBA), that use known anchor point coordinates to define the lower and upper bounds of the location of a target. These methods operate by computing the geometric intersections of range circles (in the case of the CBA) or square bounding boxes (in the case of the SBA), based on sensor data. The output is a significantly reduced feasible search region that mathematically bounds the area in which the true location lies. This modeling not only narrows the optimization domain, thereby improving computational efficiency, but also provides a centroid-based initialization point that is highly likely to be near the global minimum.

### 2.1. Square-Based Approach (SBA)

**Figure 1:**
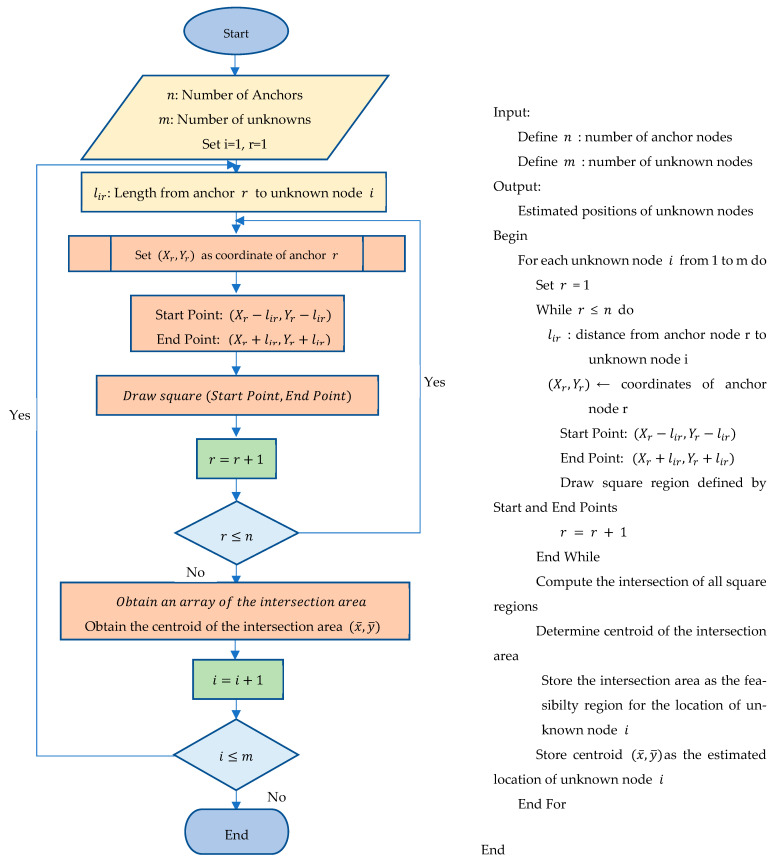
The square-based approach (SBA).

The min–max model, which is also called the square-based approach (SBA) [21,28,29], leverages the known positions of each anchor $\left(X_{r},Y_{r}\right)$ and the shortest distance $l_{ir}$ from each anchor to a target node to construct a bounding box around each anchor and the target node. This algorithm, which is illustrated in Figure 1, is initialized with the following parameters:

$l_{ir}\;$ is the minimum distance from anchor r to a node i, n is the total number of anchors in the local network, m is the total number of unknown nodes in the local network, r is the anchor counter that counts from one to n, and $\left(X_{r},Y_{r}\right)$ are the x and y coordinates of the anchor node r. u(x,y) are the estimated coordinates of node r using the min–max algorithm.

The boundary box can be constructed as follows. Starting with the distance $l_{ir}$ and an anchor with the coordinates $\left(X_{r},Y_{r}\right),$ a square can be drawn by adding and subtracting the length $l_{ir}$ from the anchor coordinates $\left(X_{r},Y_{r}\right)$ to obtain the start point $\left(X_{r}-l_{ir},Y_{r}-l_{ir}\right)$ and the end point $\left(X_{r}+l_{ir},Y_{r}+l_{ir}\right)$ of a square r. For each node, n squares are constructed, one from each anchor. The center of the intersection area $\left(\bar{x},\;\bar{y}\right)$ of the squares of one node is used as an estimate of the node position.

In this approach, the positions of unknown nodes are estimated using distances with the percentage of error from the anchor nodes with fixed coordinates. The error is estimated from the ultrasonic module’s datasheet [41] and also from the laboratory experiments performed for the real data. For each anchor, a square is drawn centered on its location, with a side length equal to twice the estimated distance to the unknown node. The area where all these squares overlap represents the region where the unknown node is most likely located. The centroid, which is the midpoint of this intersection, is the estimated position. This method offers a simple, low-complexity solution for localization, which is especially useful in systems with limited computational resources.

**Figure 2:**
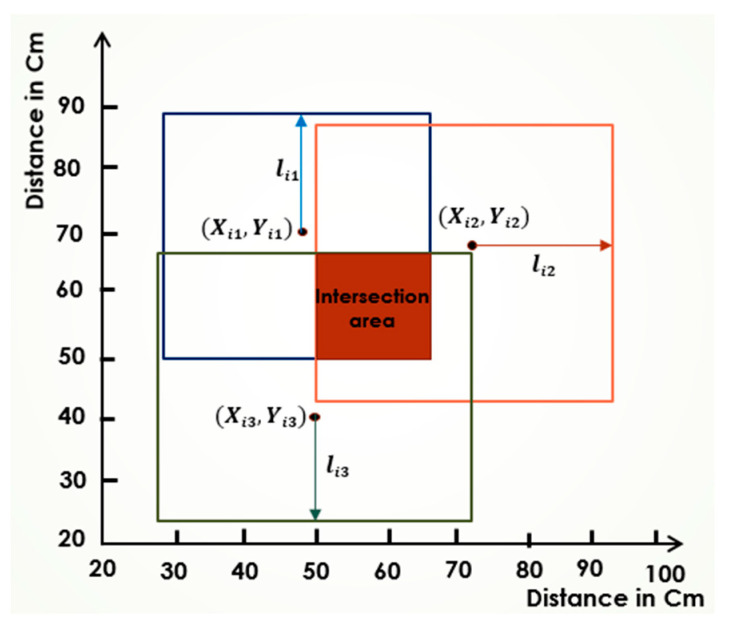
Illustration of the SBA using three anchors.

**Figure 3:**
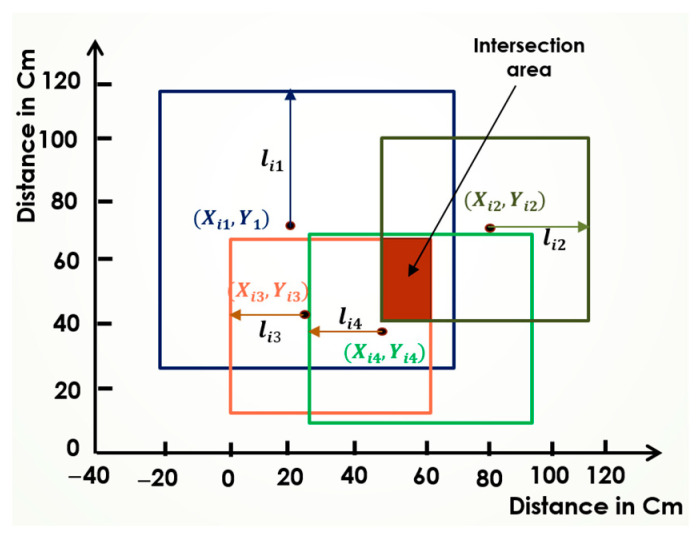
Illustration of the SBA using four anchors.

In the SBA, increasing the number of anchors directly enhances the node’s position accuracy. Figure 2 illustrates the SBA’s operation for obtaining an approximate position using three anchors, while Figure 3 shows the estimated position with four anchors. As shown, the intersection area becomes smaller with four anchors compared to three, reducing the uncertainty of the node’s position. Further increasing the number of anchors continues to minimize the intersection area, allowing for an even greater accuracy.

With a sufficiently large number of anchors, the intersection area almost becomes a single point, which closely approximates the node’s exact position. However, it also significantly increases the computational overhead and the likelihood of interference among signals, especially in noisy indoor environments. Furthermore, managing synchronization between a large number of anchors can introduce additional complexity. Therefore, an optimal number of well-placed anchors, combined with intelligent search strategies, is preferable to simply increasing the anchor density.

The intersection area is calculated through a series of steps based on the number of anchors. The process begins by determining the overlap between the squares defined by the first two anchors. For each square, the four corners are compared, selecting only the inner corners that fall within both squares. This comparison yields two points—one from each square—defining the vertices of a new rectangle that represents the intersection area.

In the next step, this new rectangle is further refined by intersecting it with the square defined by the third anchor, following the same corner comparison method to generate another rectangle. This process is repeated iteratively with each additional anchor until a final intersection rectangle is obtained. The midpoint, or centroid, of the intersection area is then calculated by averaging the x-coordinates and y-coordinates of the rectangle’s start and end points, providing an initial position estimate for further localization refinement.

### 2.2. Proposed Circular-Based Approach (CBA)

**Figure 4:**
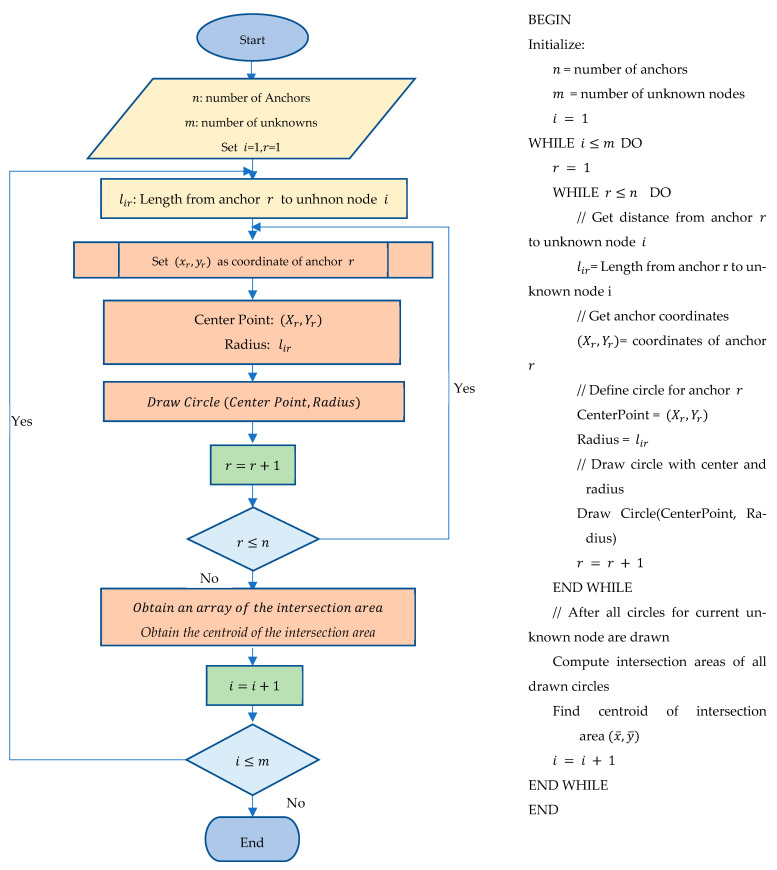
The circular-based algorithm.

The SBA offers a large search area for finding the real position, but the CBA minimizes the search area. The CBA, also known as trilateration [37], shares similarities with the SBA but utilizes circles instead of squares to define the localization area. The proposed CBA offers a new way to define the centroid. Figure 4 illustrates the sequential steps of the CBA, showing each anchor has a coordinate $\left(X_{r},Y_{r}\right)$ to a destination node, where the anchor is located at the center of the circle and the radius is the distance $l_{ir}$. The CBA is initialized with similar parameters as those used to initialize the SBA, where $l_{ir}$ is the minimum distance from anchor r to a node, and it is also the radius. For an anchor r with coordinates $\left(X_{r},Y_{r}\right)$, a circle is constructed using the distance $l_{ir}$ from the anchor to a node using (1) as follows:

(1) $${\left(x-x_{r}\right)}^{2}+{\left(y-y_{r}\right)}^{2}={l_{ir}}^{2}$$

For each node, n circles are constructed, one from each anchor. Solving for n in these equations results in an intersection area. The center of the intersection area is the estimated node position.

As illustrated in Figure 4, at least three distinct anchor nodes are required to accurately localize an object with a fixed position in a two-dimensional space. For each anchor, a circle is established, resulting in two intersection points for any pair of circles, thereby creating an ellipsoidal intersection area. When the third circle is introduced, it generates an interior curvilinear triangular intersection area, referred to as the relevant intersection area. This area is essential for estimating the object’s position.

To estimate all possible points within this relevant intersection area, a horizontal line is employed, moving downward to systematically scan the entire intersection region. This process involves forming an array of points that fall within the intersection area. For three nodes, the horizontal line scans the workspace by determining the intersections of the circles with the horizontal line through direct substitution into each equation to compute the corresponding x-coordinates, thereby identifying the points within the curvilinear triangle formed by the intersections.

In the scenario involving three circles, there will be at least four points of intersection among all the circles. Starting from the uppermost intersection point of the three circles, the scanning line moves downward in fixed increments. For each horizontal line position, the intersection points are calculated, taking into account that there will be at least four and at most six intersection points detected. If there are four intersection points, the second and third points will reside within the inner area; conversely, if there are six intersection points, the third and fourth points will lie within the inner area. Additionally, the centroid of the intersection points can be calculated, serving as an effective estimate of the object’s position within the relevant intersection area.

To illustrate this idea, a numerical example is introduced as follows:

Numerical example:

Three anchors act as the center of the three circles:

Anchor 1 at X = 50, Y = 3 with a radius of 40;

Anchor 2 at X = 2, Y = 62 with a radius of 70; and

Anchor 3 at X = 97, Y = 63.5 a with radius of 50.

The equations of the three circles are as follows:

(2) $${\left(X-50\right)}^{2}+{\left(Y-3\right)}^{2}=40^{2}$$

(3) $${\left(X-2\right)}^{2}+{\left(Y-62\right)}^{2}=70^{2}$$

(4) $${\left(X-97\right)}^{2}+{\left(Y-64\right)}^{2}=50^{2}$$

Solving the three equations together gives the intersection points shown in Table 1.

As with the SBA, increasing the number of anchors reduces the intersection area, which results in an increase in the position accuracy. The best result is obtained when the intersection area is reduced to nearly one point.

**Figure 5:**
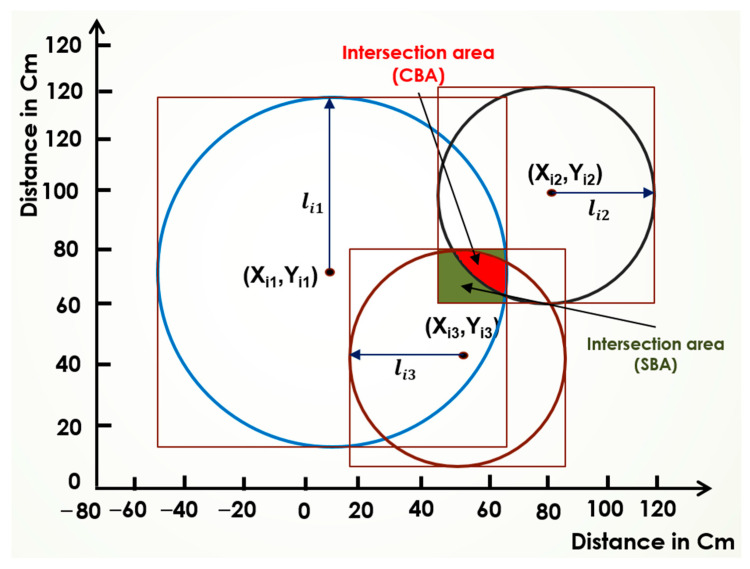
Illustration of the intersection areas of the CBA.

Figure 5 shows the difference between the intersection area when applying the SBA and the CBA algorithms. As can be seen, three anchors are used $\left(X_{1r},Y_{1r}\right),\left(X_{2r},Y_{2r}\right),(X_{3r},Y_{3r})$ with estimated lengths to the target of $l_{i1},l_{i2},l_{i3}$, respectively. The intersection area when applying the CBA algorithm is smaller compared to the area obtained when applying the SBA. Therefore, the centroid obtained from the CBA could be more accurate than that obtained from the SBA.

The SBA and CBA are used to initialize the CSA algorithm to enhance the positioning accuracy, as is explained in the next section.

## 3. Conventional Swarm Optimization Algorithms

Swarms optimization algorithms are employed to solve various types of problems [38]. This section explores the basics of the Particle Swarm Algorithm (PSA) and the Crow Search Algorithm (CSA).

These types of algorithms apply swarm optimization on the intersection area obtained from the mathematical-based approach. They work iteratively to minimize an objective function, which results in the minimization of the error in the estimated position.

### 3.1. Objective Function

The localization problem is described as an optimization task, in which the goal is to estimate the real position of unknown nodes based on the known positions of anchor nodes and the inter-node distances. Both the Particle Swarm Algorithm (PSA) and the Crow Search Algorithm (CSA) aim to minimize the following objective function [31]:

(5) $$f_{\left(n\right)}=\sum_{i=m+1}^{n}\left(\sum_{j\in N_{i}}{\left({\hat{l}}_{ij}-l_{ij}\right)}^{2}\right)$$

where ${\hat{l}}_{ij}$ is the estimated Euclidean distance between the unknown node *i* and anchor node *j*, $l_{ij}$ is the measured (or actual) distance between the two nodes, $N_{i}$ represents the set of anchor nodes, m is the number of anchor nodes, and n is the total number of nodes.

${\hat{l}}_{ij}$ is the estimated Euclidean distance between node i and an anchor j, which is found by the following [31]:

(6) $${\hat{l}}_{ij}=\sqrt{{\left({\hat{x}}_{i}-x_{j}\right)}^{2}+{\left({\hat{y}}_{i}-y_{j}\right)}^{2}}$$

where $\left({\hat{x}}_{i},{\hat{y}}_{j}\right),\left(x_{i},y_{j}\right)$ represent the coordinates of the unknown node *i* and anchor node *j*, respectively.

### 3.2. PSA

The Particle Swarm Algorithm (PSA) is a nature-inspired optimization technique that simulates the collective behavior of swarms [45], in which each particle represents a potential solution within the search space. The PSA has demonstrated strong performance across various optimization tasks [46,47]. In the context of localization, each particle explores possible object positions, and its movement is guided by both its own experience and that of neighboring particles. The algorithm evaluates each particle’s position using a predefined objective function, with the goal of minimizing localization error. The objective function used in this study is defined as follows:

(7) $$x_{i}\left(t+1\right)=\;x_{i}\left(t\right)+v_{i}\left(t+1\right)$$

where $x_{i}$, $v_{i}$ are the position and velocity of particle *i*.

Each particle is moving through the search space by adding the velocity to the current position as follows:

(8) $$v_{i}\left(t+1\right)=\;wv_{i}\left(t\right)+c_{1}r_{1}(p_{i}\left(t\right)-x_{i}\left(t\right))+c_{2}r_{2}\left(x_{i}^{g}\left(t\right)-x_{i}\left(t\right)\right)$$

w is the inertia weight controlling the influence of the previous velocity, $c_{1},c_{2\;}$ are acceleration coefficients representing the cognitive (self-learning) and social (swarm-learning) components, respectively, $r_{1},r_{1}\;$ are random numbers in the interval [0, 1], $p_{i}\left(t\right)\;$ is the best position found by particle *i*, $x_{i}^{g}\left(t\right)\;$ is the best position found by the whole swarm, and t denotes the current time (iteration).

The particles move based on a random walk. Therefore, a population of particles is initialized with random positions and velocities on the selected search space as mentioned in (7). The movement is affected by the velocity and the previous best positions (particle memory).

### 3.3. Crow Search Optimization Algorithm (CSA)

#### 3.3.1. Inspiration

The CSA is a meta-heuristic algorithm, which was introduced in [48]. The main idea is based on mimicking the behavior of crows in nature. Variant CSA versions are introduced to tune the performance of the basic CSA for different applications [46,47,48]. Many features of the CSA are related to crows. For example, crows can remember faces and distinguish between them [38]. The CSA is utilized to minimize the localization error by efficiently exploring the search space defined by the objective function in Equation (5).

#### 3.3.2. Mathematical Model

The terms are defined as follows: N is the number of crows/flock, j is a crow, D is the number of dimensions in the search space (fixed here as two) for 2D localization, t is the current iteration, $t_{max}\;$ is the maximum number of iterations, and $y_{j}\left(t\right)\;$ is the memory position (best-known position) of crow *j* at iteration *t*.

Each crow has a position which updates iteratively according to its awareness of being tracked and its memory of successful locations. The algorithm evaluates each crow’s position according to the objective function $f_{\left(n\right)}$ and updates its locations accordingly to minimize the localization error. The generic position update is defined as follows:

(9) $$y_{j}\left(t+1\right)=\left\{\begin{array}{l} y_{j}\left(t\right)+r\;x\;{fl}_{j}\left(t\right)\;x\;(N_{z}\left(t\right)-y_{j}\left(t\right)),\;\;\;\;Crow\;j\;unaware\;to\;be\;followed\\ \;\;\;\;\;\;\;\;\;\;\;\;\;Choose\;a\;random\;position,\;\;\;\;\;\;\;\;\;\;else \end{array}\right.$$

where $y_{j}\left(t\right)$ is the position of crow j at iteration t, r is a random number in the interval [0, 1], and ${fl}_{j}\left(t\right)$ is the flight length parameter controlling the step size.

Case 1: Crow j follows crow z without being detected.

In this case, the new position of crow j is calculated as follows:

(10) $$y_{j}\left(t\right)+r\;x\;{fl}_{j}\left(t\right)\;x\;(N_{z}\left(t\right)-y_{j}\left(t\right))$$

The crows search the intersection area depending on the objective function. The main aim is to minimize the objective function to minimize error.

Case 2: Crow z knows it is being tracked. To avoid being tracked, the crow moves to a random position within the feasible space:

(11) $$y_{j}\left(t+1\right)=rand(D)$$

where $rand\left(D\right)$ generates a random position in the D-dimensional search space.

The CSA mimics crows’ behavior in nature, according to the previous equations, to search for the real position within the constrained area. Moreover, the objective function for the localization problem is used to minimize the error obtained from each anchor.

## 4. Proposed Modified Crow Search Algorithm (MCSA)

The algorithm aims to minimize the distance error by simulating the behavior of crows searching for a solution, depending on the area and starting from the centroid point obtained from the mathematical approaches. The mathematical approaches minimize the search area and, starting from the centroid as the starting point, enhance the results obtained, considering both individual exploration and social learning.

The MCSA shown in Algorithm 1 begins with the initialization of positions and memories, in which each crow in the flock is assigned a feasible solution vector obtained from the mathematical model. This initial position is stored as its memory. Next, the fitness of each crow is evaluated using a predefined fitness function. In the subsequent step, each crow randomly selects another crow and adjusts its position based on the selected crow’s memory; the algorithm in Equation (9) is modified in the CSA to the following:

(12) $$y_{j}\left(t+1\right)=\left\{\begin{array}{l} y_{j}\left(t\right)+r\;x\;{fl}_{j}\left(t\right)\;x\;(N_{z}\left(t\right)-y_{j}\left(t\right)),\;\;\;\;Crow\;j\;unaware\;to\;be\;followed\\ \;\;\;\;\;\;\;\;\;\;\;\;\;\;Starting\;from\;centroid\;(\bar{x},\;\bar{y})\;\;\;\;else \end{array}\right.$$

Case 1: Crow j follows crow z without being detected.

In this case, the new position of crow j is calculated as follows:

(13) $$y_{j}\left(t\right)+r\;x\;{fl}_{j}\left(t\right)\;x\;(N_{z}\left(t\right)-y_{j}\left(t\right))$$

where $y_{j}\left(t\right)$ is the position of crow j at iteration t, r is a random number in the interval [0, 1], and ${fl}_{j}\left(t\right)$ is the flight length parameter controlling the step size.
**Algorithm 1:** Modified Crow Search Algorithm (MCSA)
**//Problem Initialization** ${OBj}_{\left(F\right)}:$ The objective function(minimize the distance error) ${Fit}_{\left(F\right)}$: The fitness Function ${Deci}_{Var}$: Decision variable ***l_ir_***
${FL}_{size}$: The flock size ${Max}_{itr}$: maximum number of iterations $x^{i,iter}$: A vector for the position of crow i at time iter in the search space obtained from the mathematical approach ${F_{Len}(i)}^{itr}$: The length of flight of crow i in specific iteration P(Åw): The probability of awareness ${Crow}_{num}$: Number of crows of the flock ${FS}_{vector}$: The feasible solution vector(the intermediate area obtained from the CBA) $r_{i}$: A random number with uniform distribution between 0 and 1 ${Feasb}_{TH}:The\;feasability\;threshold$ **1. Positions and memories Initialization**          For Each i in ${Crow}_{num}$          Generate (${FS}_{vector}$)          Fill(${Pos}_{initial}⟶Memory$) **//2. Fitness function Evaluation**          Calculate (${Fit}_{\left(F\right)}$) **//3. New position Generation**          For Each i in ${Crow}_{n}$          Rand_Select (j$\in {Crow}_{num}$)          If $r_{j}$ > ${P(Åw)}_{j}$           Generate $\left({New}_{Pos}\right)$          $x^{i,iter+1}=x^{i,iter}+r_{j}\times {F_{Len}(i)}^{itr}\times \left(m^{j,iter}-x^{i,iter}\right)$          else Generate $\left({Rand}_{Pos}\right)$ **//4. Check feasibility of new positions**          For Each i in ${Crow}_{num}$          Check-Feasibility $\left({New}_{Pos}\right)$          If ${New}_{Pos}$
≥
${Feasb}_{TH}$                ${New}_{Pos}⟶Update(Pos)$          else No-Update(Pos) **//5. Evaluate fitness function for new positions**          For Each i in ${Crow}_{num}$          Calculate (${Fit}_{\left(F\right)}$) **//6. Update memory**          For Each i in ${Crow}_{num}$          If ${{Fit}_{\left(F\right)}}_{i+1}>{{Fit}_{\left(F\right)}}_{i}$          ${New}_{Pos}⟶Update(Pos)$          Else No-Update(Pos) **//7. Check termination criterion**          If i $\leq {Max}_{itr}$ Repeat step 1

The crows search the intersection area obtained from the mathematical approaches depending on the objective function. The main aim is to minimize the objective function to minimize error.

Case 2: The algorithm is updated to obtain a more accurate position with the minimum number of iterations starting from the centroid as follows:

(14) $$y_{j}\left(t+1\right)=(\bar{x},\;\bar{y})\;$$

From the mathematical approaches, the centroid is the most powerful starting point to obtain the real position. Moreover, the mathematical approaches minimize the search area, and the new positions are then checked for feasibility. If a position meets the feasibility criteria in (8), it is accepted; otherwise, the previous position is retained. The fitness of these new positions is then recalculated. If a crow’s new position has better fitness than its previous one, the memory is updated with the new position; if not, the memory remains unchanged. This process—generating new positions, evaluating fitness, and updating memories—continues iteratively until the termination criteria (8) are met.

The objective function ${Obj}_{\left(f\right)}$ aims to minimize the distance error between the estimated and actual positions of unknown nodes, the fitness function ${Fit}_{\left(f\right)}$ is used to evaluate how well a candidate solution satisfies the objective function, flock size ${FL}_{size}$ is the total number of crows (agents) used in the search algorithm, and ${Max}_{itr}$ is the maximum number of iterations allowed during the optimization process.

## 5. Performance Evaluation

Two groups of experimental tests are conducted to evaluate the effectiveness of the the CBA-PSA and CBA-CSA, and they are compared with the SBA and the CBA. The first group of tests uses real data, while the second group uses simulated data and is applied on a larger area compared to the first group. Each test is applied on 50 different positions, where each position is used to obtain 50 different runs, resulting in a total of 2500 runs.

**Figure 6:**
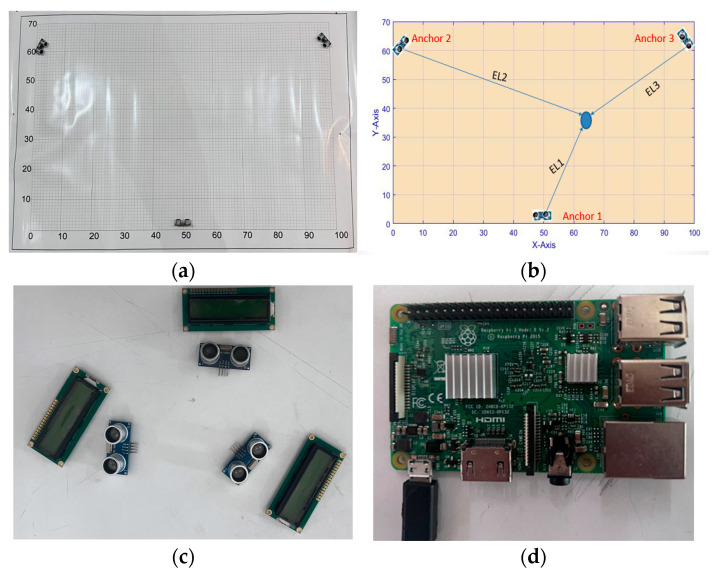
(**a**,**b**) Setup area and estimating the distance to a node; (**c**,**d**) Hardware components.

Case 1—Real data: Figure 6 shows the setup and hardware used. An area of 100×70 cm is used as shown in Figure 6a,b. The test uses three ultrasonic modules (Ultrasonic sensor HC-SR04), three LCDs (to show the three distances obtained from each module) as shown in Figure 6c, and Raspberry Pi as shown in Figure 6d. The test is performed as follows.

(1) Establish a platform with a scale of 100×70 cm. Afterwards, set up three anchors using the three ultrasonic modules on the scaled platform. The coordinates of the three anchors are as follows:
  Anchor1 at x = 50, y = 2.5. Anchor2 at x = 2, y = 61.5. Anchor3 at x = 97.5, y = 63.5;
(2) Set a node in the “in-between” area and record its real position (for error calculation). Estimate the distance to the node using the three anchors. The estimated distances are defined as EL1, EL2, and EL3 as shown in Figure 6b;
(3) Estimate the position of the node as the centroid of the intersection area using the SBA by drawing three rectangles using the obtained three lengths of EL1, EL2, and EL3;
(4) Estimate the position (the centroid of the intersection area) of the node using the CBA, with the three lengths of EL1, EL2, and EL3 representing the radii of the three circles.

The same steps are repeated 50 times for each of the 50 different positions to obtain 2500 results.

The conventional PSA and CSA and the proposed MPSA and MCSA are tested. The testing is performed as follows: (1) Track a node once using the PSA and again using the CSA; and (2) perform the CBA with 20 search agents (particles or crows depending on the selected algorithm) and 50 iterations to obtain the fitness function by using the three anchors of AN1, AN2, and AN3 and the obtained three lengths of EL1, EL2, and EL3, as indicated in Figure 6.

**Figure 7:**
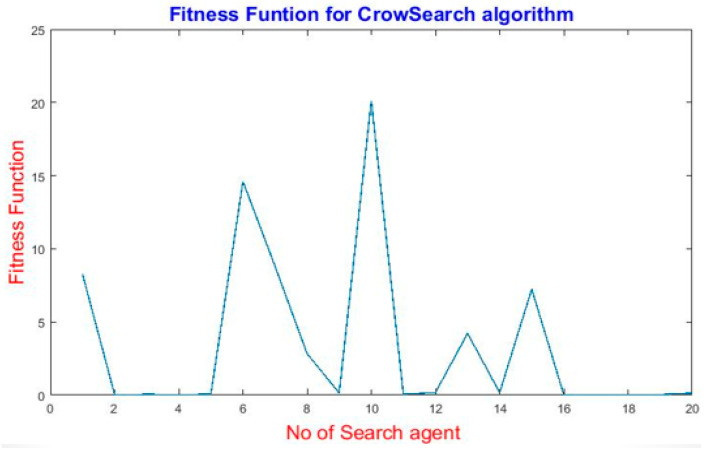
The fitness function for MCSA.

Figure 7 shows the fitness function values as a function of the number of search agents. The fitness function represents the quality of solutions found by the algorithm for a given number of agents. Lower fitness values usually indicate better solutions in minimization problems. The fitness function shows a non-linear and highly variable pattern as the number of agents increases. Peaks at certain numbers of agents (e.g., 6, 10, and 15) indicate poorer solutions (higher fitness values). Minimal fitness function values (e.g., around agents 2–4, 8, 12, and 17–20) suggest better-performing configurations.

**Figure 8:**
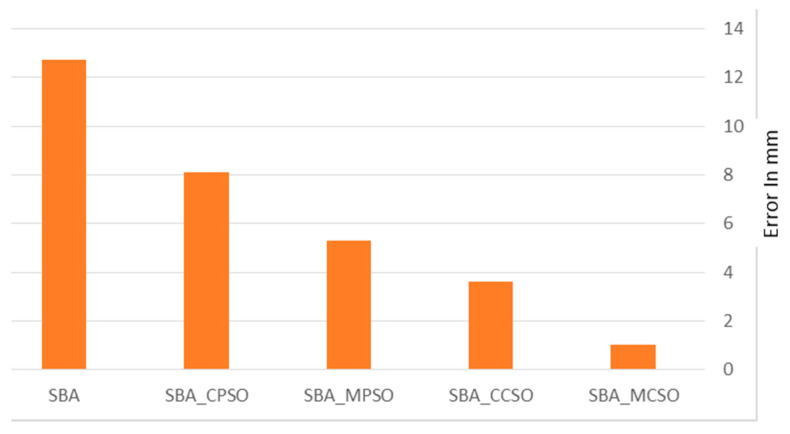
RMS for the SBA with PSA, MPSA, CSA, and MCSA.

The Root Mean Square Error (RMSE) is calculated for the PSA and CSA using the SBA. Table 2 and Figure 8 show the results. The units are expressed in millimeters (mm). As can be seen, the results obtained from the SBA and CBA have large errors compared to those obtained using the SBA_PSA or SBA CSA. Moreover, the CSA gives more accurate results compared with those obtained from the PSA. The SBA_MCSA performs the best with the lowest RMSE of 1.0112 mm, indicating the highest accuracy among all the methods.

The RMSE values are shown as percentages relative to the worst-performing model (which is the SBA with an RMSE = 12.704553 mm). The percentage improvement of each algorithm over the SBA is as shown in Table 3.

As can be seen in Table 3, the SBA_MCSA gives the most significant improvement, reducing the RMSE by approximately 92%, demonstrating a substantial enhancement in accuracy. Meanwhile, the SBA_CSA, SBA_MPSA, and SBA_PSA reduce the RMSE by 71%, 58%, and 36%, respectively. These results clearly indicate that integrating the MCSA into the SBA framework yields the most accurate outcomes.

**Figure 9:**
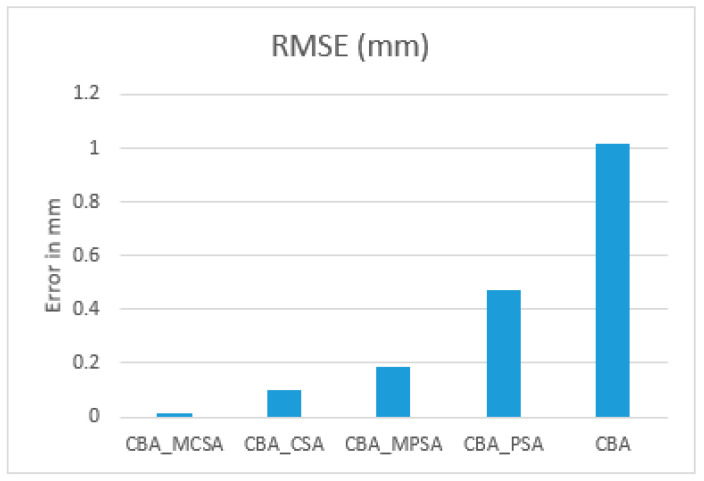
RMS for the CBA with PSA, MPSA, CSA, and MCSA for real data.

The RMSE for the PSA and CSA using the CBA is shown in Table 4 and Figure 9. The results are expressed in mm. As can be seen, the results obtained from the CSA have a larger error than that obtained using the SBA with PSA or the CBA with CSA. Moreover, the CSA gives more accurate results compared to those obtained from the PSA.

The RMSE values as percentages are shown relative to the worst-performing model (which is the CBA with an RMSE = 1.0161886 mm). The percentage improvement of each method over the CBA is shown in Table 5.

As can be seen in Table 5, the CBA_MCSA gives the most significant improvement, reducing the RMSE by approximately 98.97%, demonstrating an enhancement in accuracy. The CBA_CSA follows with a 90.34% improvement, and the CBA_MPSA shows an 81.91% reduction in error. The CBA_CPSA achieves a more modest improvement of 53.46%. These results clearly indicate that integrating the MCSA into the CBA algorithm yields the most accurate outcomes.

The results obtained from Table 2 and Table 4 indicate that the CBA is more accurate than the SBA as the intersecting area is smaller and the obtained centroid is more accurate. Moreover, using the CBA with the MPSA and MCSA shows that the MCSA has a better performance and gives results that are more accurate.

Case 2—Simulated Data for area of 1400 m×400 m

The previous tests are repeated for a simulated platform of 1400 m×400 m with 50 mobile node positions, in which each node is evaluated in 50 runs to obtain a total of 2500 runs.

The ultrasonic sensor operates at a frequency of 40 kHz, which is a standard frequency for most commercial ultrasonic transceivers used in localization applications. This frequency provides a good balance between range, accuracy, and environmental robustness for indoor environments.

The expected signal-to-noise ratio (SNR) in ultrasonic localization systems varies depending on environmental conditions (e.g., surface reflections, ambient noise, temperature, and humidity). However, in controlled indoor environments, the typical SNR for ultrasonic signals ranges from 20 dB to 40 dB. In our experiments, the system was evaluated under moderate noise conditions, and the effective SNR was maintained around 30 dB. This SNR level is sufficient to ensure reliable time-of-flight measurements and accurate distance estimation, which is critical for the performance of the proposed localization algorithm.

In this case, the estimated distance could be obtained by adding an error to the real distance as follows [39]:

(15) $${El}_{i}=L_{i}+e_{i}$$

where $e_{i}=\varepsilon_{i}+b;\;\varepsilon_{i}=N\left(0,\sigma_{i}^{2}\right);$ $\varepsilon_{i}$ is the independent form of $\varepsilon_{j}$ where i≠j; (i,j∈{1,………..M}); and b is the synchronization bias. Therefore, the standard deviation $\sigma_{i}$ for the estimated error distance between anchor i and a node could be obtained as a linear equation of the real distance between them as follows [39]:

(16) $$\sigma_{i}=0.01L_{i}+0.08$$

The constants for this equation are obtained by using a channel model as in [41] and the energy detection receiver [49,50] by a band-pass filter followed by a square law device and an integrator.

**Figure 10:**
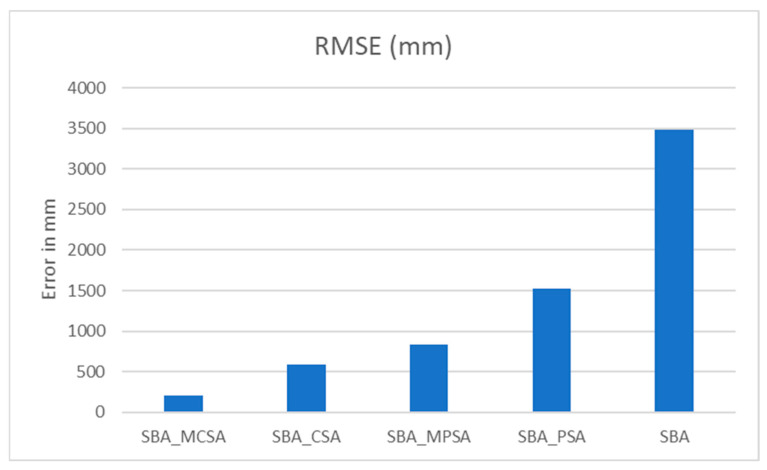
RMSE for the CBA with PSA, MPSA, CSA, and MCSA.

The RMSE measured in mm for the PSA and CSA using the SBA is shown in Table 6 and Figure 10. As can be seen, the results obtained from the SBA have a large error compared to those obtained using the SBA with PSA or the SBA with CSA. Moreover, the CSA gives more accurate results compared to those obtained from the PSA.

As shown in Table 7, when comparing the RMSE values as percentages relative to the base SBA model, the SBA_MCSA results in the most significant improvement, reducing the RMSE by approximately 94% and demonstrating an enhancement in accuracy. The SBA_CSA follows with an 83% improvement, and the SBA_MPSA shows a 76% reduction in error. The SBA_CPSA achieves a more modest improvement of 56%. These results clearly indicate that integrating the MCSA into the SBA framework yields the most accurate outcomes.

**Figure 11:**
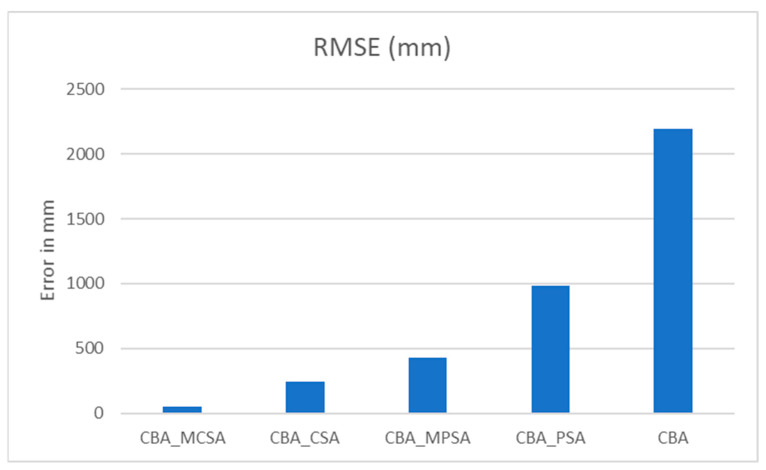
RMSE for the CBA with PSA, MPSA, CSA, and MCSA for simulated data.

The RMSE measured in mm for the PSA and CSA using the CBA is shown in Table 8 and Figure 11.

As shown in Table 9, when comparing the RMSE values as percentages relative to the base CBA model, the CBA_MCSA results in the most significant improvement, reducing the RMSE by approximately 98%, demonstrating a substantial enhancement in accuracy. The CBA_CSA follows with a 90% improvement, and the CBA_MPSA shows an 82% reduction in error. The CBA_CPSA achieves a more modest improvement of 53%. These results clearly indicate that integrating the MCSA into the CBA framework yields the most accurate outcomes, with a progressive degradation in performance observed in the other variants.

**Figure 12:**
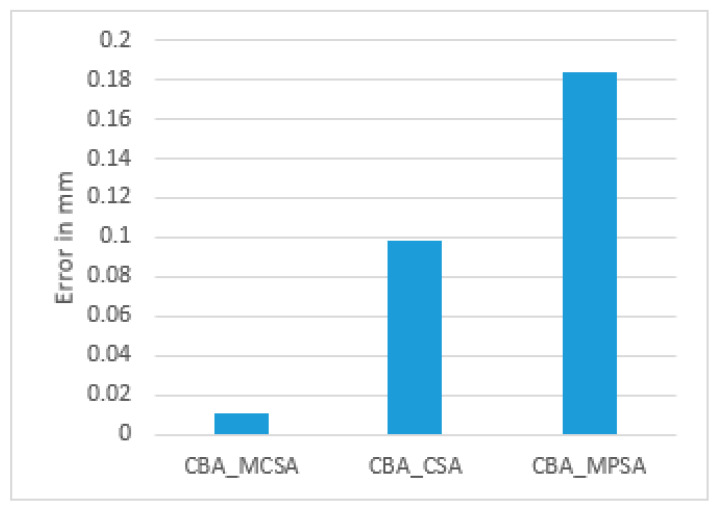
RMSE for the PSA, CSA, and LSM.

As can be seen, the results obtained from using the mathematical algorithm only (circle) have a large degree of error compared to those obtained using the SBA with PSA or the CBA with CSA. Moreover, the CSA gives more accurate results compared to those obtained with the PSA. Moreover, the obtained results from the CSA were also compared using the least squares method. The results show that using the CSA with the CBA gives more accurate results for the 2500 runs using three anchors, as indicated in Table 10 and Figure 12.

Comparing the results obtained, it can be observed that the CSA algorithm with CBA is more accurate than the PSA-based algorithm when locating a node within a small area like Case (1) or a large area like Case (2).

The comparative analysis in Table 11 highlights a broad spectrum of 2D localization techniques.

The following are compared: accuracy, infrastructure requirements, computational complexity, and real-time performance.

Traditional approaches such as 2D LiDAR with correlative SLAM [42] maintain high accuracy (95%) and real-time performance. However, they require costly and bulky LiDAR sensors, making them less practical for lightweight or cost-sensitive applications. Similarly, visual deep learning methods like LASER [41] and NeRF-based approaches [46] show strong performance (90–92%) in terms of precision. However, these methods are constrained by their reliance on GPU-accelerated processing and their high computational demands, limiting their scalability in embedded or real-time systems.

Low-cost alternatives such as magnetic field mapping [43] and smartphone sensor fusion [44] offer greater accessibility and ease of deployment. However, these methods generally suffer from reduced localization precision (65–80%) and may be susceptible to environmental noise or sensor drift. BLE/Wi-Fi-based localization methods enhanced with GNNs [45] strike a balance by offering moderate accuracy (75–85%) with the infrastructure already present in many indoor environments, although latency and calibration remain challenges.

In contrast, the proposed methodology which combines mathematical modeling with the Crow Search Algorithm achieves the highest accuracy (98%) among all surveyed methods, while preserving real-time operability and maintaining low computational complexity. This is achieved through the intelligent optimization of node positioning using a biologically inspired metaheuristic and leveraging ultrasonic modules, which are both low-cost and widely applicable in indoor environments. The approach demonstrates strong potential for practical, scalable deployments, particularly in scenarios in which accuracy and simplicity are both critical.

## 6. Summary and Conclusions

This paper introduces an intelligence-based algorithm aimed at solving the localization problem in mobile applications that utilize ultrasonic sensors. The algorithm incorporates mathematical methods—namely the square-based algorithm (SBA) and circular-based algorithm (CBA)—to define a reduced search area and determine an initial point, referred to as the centroid, for use in advanced optimization techniques.

The comparative analysis reveals that, with a minimal number of anchors, the circular-based algorithm (CBA) demonstrates superior effectiveness compared to the square-based algorithm (SBA). Consequently, the CBA emerges as a more advantageous choice for initializing swarm optimization algorithms. The performance of the algorithms is evaluated across two distinct scenarios, with results indicating that the CBA integrated with the Modified Crow Search Optimization (MCSA) algorithm yields the fewest localization errors (53.710358 mm).

According to the comparative analysis of the broad spectrum of 2D localization techniques including factors like accuracy, infrastructure requirements, computational complexity, and real-time performance, the proposed methodology (CBA_MCSA), which combines mathematical modeling with the modified Crow Search Algorithm, achieves the highest accuracy (98%) among all the surveyed methods.

Based on the findings presented, it can be concluded that the proposed circular-based algorithm, in conjunction with the modified Crow Search Optimization Algorithm, proves to be effective in solving the localization problem for networks characterized by a limited number of anchors. This approach also exhibits robustness in situations in which data from some anchors may be missing.

## Figures and Tables

**Table 1:** Three circles’ intercepted regions and centroids obtained.

Circles from intersection points of (2), (3)	(11.37, −7.37) (68.01, 38.71)
Intersection points of Circles 2–4	(51.64, 42.97) (88.23, 14.78)
Intersection points of Circles 3–4	(61.37, 99.08) (62.88, 27.45)
Lapping segments’ points	(68.01, 38.71) (51.64, 42.97) (62.88, 27.45) Lapping area contains 3 segments and triangle
Total intersection points	6
Centroid point $\left(\bar{x},\;\bar{y}\right)$	(65.445, 35.21)

**Table 2:** RMSE for real data for a 100×70 cm environment using the square-based algorithm.

Algorithm	SBA_MCSA	SBA_CSA	SBA_MPSA	SBA_PSA	SBA
RMSE (mm)	1.01124635	3.622305	5.28482251	8.087234	12.704553

**Table 3:** Percentage of RMSE for real data for a 100×70 cm environment using the square-based algorithm.

Method	RMSE (mm)	% Improvement over SBA
SBA_MCSA	1.0112	92.05%
SBA_CSA	3.6223	71.49%
SBA_MPSA	5.2848	58.42%
SBA_PSA	8.0872	36.36%
SBA	12.7046	0.00%

**Table 4:** RMSE for real data for a 100×70 cm environment using circular-based algorithms.

Algorithm	CBA_MCSA	CBA_CSA	CBA_MPSA	CBA_PSA	CBA
RMSE (mm)	0.010456	0.0982047	0.183823	0.47290876	1.0161886

**Table 5:** Percentage of RMSE for real data for a 100×70 cm environment using the circle-based algorithm.

Algorithm	RMSE (mm)	% Improvement over CBA
CBA_MCSA	0.010456	98.97%
CBA_CSA	0.0982047	90.34%
CBA_MPSA	0.183823	81.91%
CBA_CPSA	0.47290876	53.46%
CBA	1.0161886	0.00%

**Table 6:** RMSE for real data for a 1400 m×400 m environment using rectangle-based algorithms.

Algorithm	SBA_MCSA	SBA_CSA	SBA_MPSA	SBA_PSA	SBA
RMSE (mm)	202.316228	583.0913	835.8246	1522.487	3483.753

**Table 7:** Percentage of RMSE for real data for a 1400 m×400 m environment using rectangle-based algorithms.

Algorithm	RMSE (mm)	% Improvement over SBA
SBA_MCSA	202.3162	94.19%
SBA_CSA	583.0913	83.26%
SBA_MPSA	835.8246	76.01%
SBA_PSA	1522.487	56.30%
SBA	3483.753	0.00%

**Table 8:** RMSE for real data for a 1400 m×400 m environment using circle-based algorithms.

Algorithm	CBA_MCSA	CBA_CSA	CBA_MPSA	CBA_PSA	CBA
RMSE (mm)	53.710358	243.8709	427.8038	986.2809	2194.176

**Table 9:** Percentage of improvement for real data for a 1400 m×400 m environment using circle-based algorithms.

Algorithm	RMSE (mm)	% Improvement over CBA
CBA_MCSA	53.7104	97.55%
CBA_CSA	243.8709	88.89%
CBA_CPSA	427.8038	80.51%
CBA_PSA	986.2809	55.05%
CBA	2194.176	0.00%

**Table 10:** RMSE for real data for a 1400 m×400 m environment using CBA (PSA, CSA, LSM).

Algorithm	MCSA	MPSA	LSM
RMSE (mm)	53.710358	427.8038	1004.206

**Table 11:** A comparison between the proposed methodology and recently published articles on the state of the art of node localization.

Key Reference(s)	Technique	Accuracy (Relative %)	Real-Time	Infrastructure Requirement	Learning-Based	Complexity
Zhao et al. [40]	PNeRFLoc (Visual + NeRF)	92%	Limited	Camera + GPU	Yes	Very High
Min et al. [41]	LASER (Visual DL-based)	90%	No	Camera	Yes	High
Du et al. [42]	2D LiDAR + Correlative SLAM	95%	Yes	LiDAR scanner	No	Medium
Sensors and Actuators [43]	Magnetic Field Mapping	65%	Yes	Magnetometer only	No	Low
Sensors [44]	Smartphone Sensor Fusion	70–80%	Yes	IMU + phone sensors	No	Medium
Aziz & Koo [45]	BLE or Wi-Fi + GNN	75–85%	Moderate	BLE/Wi-Fi + DB	Yes	Medium–High
Liu et al. [46]	NeRF-Loc	90%	Limited	Camera + GPU	Yes	Very High
Proposed Methodology	Mathematical Modeling and Crow Search Algorithms	98%	Yes	Ultrasonic modules	yes	low

## Data Availability

The original contributions presented in this study are included in the article. Further inquiries can be directed to the corresponding author.

## Abbreviations

The following abbreviations are used in this manuscript:

SBA	Square-based approach
CBA	Circular-based approach
PSA	Particle Swarm Algorithm
CSA	Crow Search Algorithm
MCSA	Modified Crow Search Algorithm
SBA_MCSA	Square-based approach with Modified Crow Search Algorithm
CBA_MCSA	Circular-based approach with Modified Crow Search Algorithm
